# The Effects of Isometric Fatigue on Trunk Muscle Stiffness: Implications for Shear-Wave Elastography Measurements

**DOI:** 10.3390/s22239476

**Published:** 2022-12-04

**Authors:** Rok Vatovec, Žiga Kozinc, Matej Voglar

**Affiliations:** 1 Department of Physiotherapy, Faculty of Health Sciences, University of Primorska Polje 42, SI-6310 Izola, Slovenia; 2 Department of Kinesiology, Faculty of Health Sciences, University of Primorska Polje 42, SI-6310 Izola, Slovenia; 3 Andrej Marušič Institute, University of Primorska, Muzejski trg 2, SI-6000 Koper, Slovenia

**Keywords:** low back pain, low back disorders, shear modulus, ultrasound elastography, spine stiffness

## Abstract

Muscle stiffness has been implicated as a possible factor in low back pain risk. There are few studies on the effects of isometric fatigue on the shear modulus of trunk muscles. This study aimed to investigate the effects of trunk isometric fatigue on the passive and active (during low and high-level contractions) shear moduli of the erector spinae (ES) and superficial and deep multifidus (MF) muscles. We assessed passive and active shear modulus using shear-wave elastography in healthy young participants (*n* = 22; 11 males, 11 females), before and after an isometric trunk extension fatigue protocol. Maximal voluntary force decreased from 771.2 ± 249.8 N before fatigue to 707.3 ± 204.1 N after fatigue (−8.64%; *p* = 0.003). Passive shear modulus was significantly decreased after fatigue in the MF muscle (*p* = 0.006–0.022; Cohen’s d = 0.40–46), but not the ES muscle (*p* = 0.867). Active shear modulus during low-level contraction was not affected by fatigue (*p* = 0.697–0.701), while it was decreased during high-level contraction for both muscles (*p* = 0.011; d = 0.29–0.34). Sex-specific analysis indicated the decrease in ES shear modulus was significant in males (*p* = 0.015; d = 0.31), but not in females (*p* = 0.140). Conversely, the shear modulus in superficial MF had a statistically significant decrease in females (*p* = 0.002; d = 0.74) but not in males (*p* = 0.368). These results have important implications for further investigations of the mechanistic interaction between physical workloads, sex, muscle stiffness (and other variables affecting trunk stability and neuromuscular control), and the development/persistence of low back pain.

## 1. Introduction

Low back pain (LBP) remains a common problem, especially in the working-age population [1]. Although numerous studies have been conducted to determine the causes of LBP, the underlying pathology or source of pain can only be identified in a small number of cases [2,3]. While a majority of LBP cases resolve in a short time, some patients develop chronic or recurrent LBP, which is associated with impaired quality of life [4,5] and large healthcare expenses [6,7]. There is general agreement that LBP is a complex disorder with multiple risk factors and possible mechanisms leading to pain and disability [2,8,9].

In recent years, shear-wave elastography (SWE) has emerged as a tool for direct assessment of tissue stiffness [10,11]. Briefly, SWE is based on the generation of an acoustic shear wave and subsequent tracking of its propagation velocity, where the velocity is higher in stiffer tissue. Then, the shear modulus is calculated as an index of muscle stiffness [10,12,13]. The reliability of SWE to assess muscle stiffness has been confirmed, including for trunk muscles [14]. Subsequently, several studies have observed higher stiffness (shear modulus) of trunk extensors in LBP groups compared to asymptomatic controls [15,16,17]. However, no cause-effect relationship can be inferred from these case-control studies. To obtain information on mechanistic relationships between specific occupational workloads and LBP, previous studies have examined trunk mechanical and neuromuscular characteristics before and after work shifts [18,19] or simulations thereof [20,21]. Of particular interest was prolonged work in a flexed posture, such as seen in crane operators [22].

Previous studies reported possible changes in sitting balance, trunk reflex functions, and intrinsic trunk stiffness after exposure to spinal flexion [20,21,23,24], providing indications for possible mechanisms underlying the development of LBP. However, apart from preliminary evidence of increased shear modulus after prolonged sitting [19,25], the effect of prolonged flexion and other physical workloads on intrinsic trunk muscle stiffness (shear modulus) is largely unknown. Therefore, studies to examine the changes in shear modulus after prolonged flexion and physically demanding work in general are needed. However, a potential caveat for such studies is the possible influence of muscle fatigue on shear modulus. Indeed, studies examining lower limbs have shown, albeit not consistently for all muscles, a marked decrease in passive (during rest) and active (during contraction) shear modulus after isometric fatiguing protocol [26,27,28]. On the other hand, a possible increase has been noted for the passive shear modulus of the multifidus (MF) muscle [29]. Nevertheless, the effects of fatigue on trunk muscle stiffness should be further investigated. For once, increased stiffness of the superficial but not the deep multifidus have been reported in LBP groups [16], warranting a separate assessment of each layer. In addition, the passive and active shear moduli, as well as the difference between them (i.e., the contraction ratio), might play a different role (or no role) in the development and persistence of LBP [15,16]. Similarly, both active shear modulus and contraction ratio in thigh muscles have been suggested as factors in patellofemoral pain [30]. In sport science research, emerging evidence points to the role of passive and active shear modulus in rate of force development [31] and performance in tasks, such as sprinting [32] and jumping [33,34]. While shear modulus is apparently associated with muscle performance [31,32,33] and could play a role in LBP development [15,16], the effect of neuromuscular fatigue on shear modulus of back musculature is currently unknown. It has been indicated that the deterioration of trunk sensorimotor control after prolonged flexion is mainly caused by tissue creep deformation and not fatigue [35]. However, researchers also need to be aware of the possible confounding effect of fatigue on shear modulus when studying the effects of physically demanding work.

As described above, there are few studies on the effects of isometric fatigue on the shear modulus of trunk muscles. Therefore, the aim of the present study was to investigate the effects of trunk isometric fatigue on the passive and active shear moduli of the erector spinae and multifidus muscles. We hypothesized isometric fatigue will result in a decrease in passive and active shear modulus. The magnitude and between-participant variability of this decrease will provide important information for further studies examining the effects of physically demanding work on intrinsic muscle stiffness and its relationship to LBP development. As a secondary aim, we also investigated if a warm-up affects passive shear modulus.

## 2. Materials and Methods

### 2.1. Participants

The study sample included 22 participants (11 males, 11 females). The sample size was calculated at a statistical power of 0.80 and statistical significance of α = 0.05. Although very large effect sizes were reported in previous studies that investigated the effects of fatigue on shear modulus in lower limb muscles [26], our pilot measurements (*n* = 5) indicated a moderate effect size (d = 0.6). Inclusion criteria were age between 20 and 40 years and regular engagement in physical activity (>150 min/week). Participants were excluded if they reported an episode of acute LBP in the past six months, history of chronic LBP or surgery on the spine, pelvis, or hip, and in case of regular participation in sports training. The study was conducted according to the Helsinki declaration and was approved by the Slovenian National Medical Ethics Committee (approval number: 0120-234/2022/6).

### 2.2. Study Protocol

**Figure 1:**
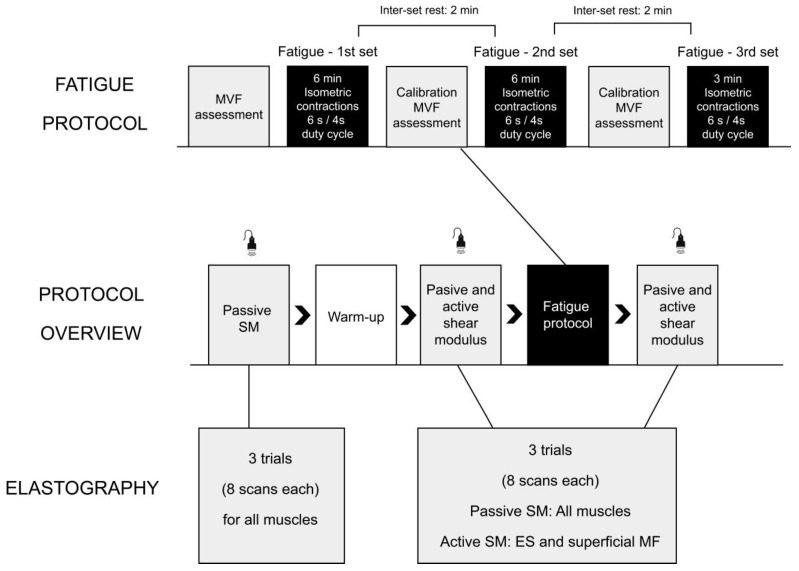
The overview of the protocol.

Upon arrival, the participants were informed about the study purpose and gave informed consent for participation. The participants completed a standardized warm-up, which consisted of 6 min of stepping on a 20-cm stepper and 10 repetitions of squats, single-leg deadlifts, lunges, push-ups, and long lever bridges. Then, baseline shear modulus values for MF and erector spinae (ES) muscles were measured. Specifically, we assessed passive shear modulus, as well as active shear modulus, during low-level and high-level submaximal contractions (described in later paragraphs), always in this order. Subsequently, participants were exposed to an isometric trunk extension protocol, after which the passive and active shear moduli were re-evaluated again in the same order. As a secondary aim, we assessed if the warm-up had any effects on passive shear modulus. Therefore, passive shear modulus was also assessed before the warm-up. The detailed protocol is shown in Figure 1 and explained in the following paragraphs.

### 2.3. Shear-Wave Elastography

**Figure 2:**
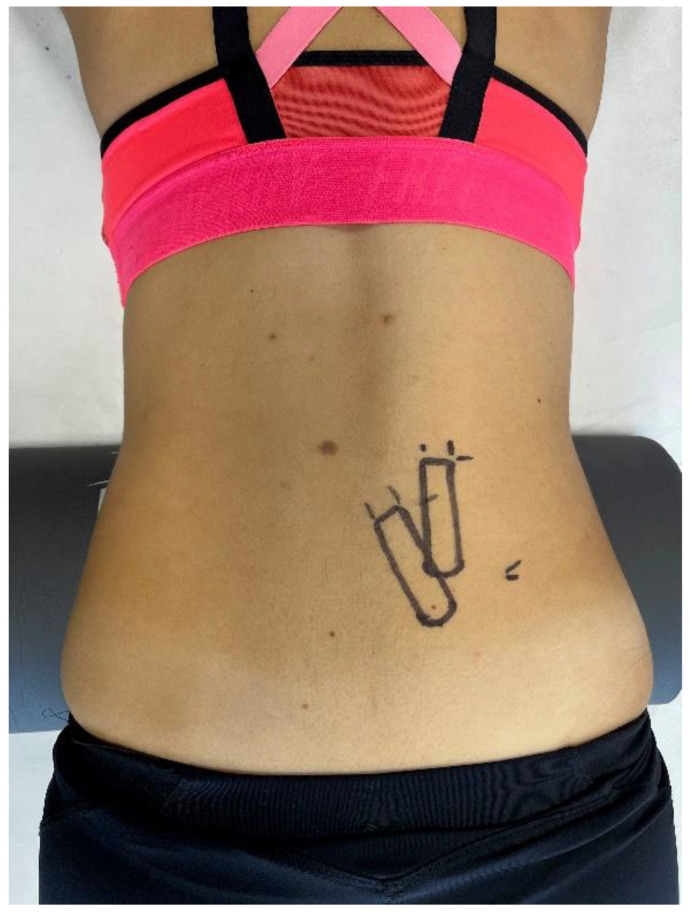
Example of marks drawn with semi-permanent marker.

Shear modulus was measured with SWE using Resona 7 ultrasound (Mindray, Shenzhen, China). Prior to the first measurement, the probe location was marked using a semi-permanent marker based on a previously published reliability study by Koppenhaver et al. [14]. The muscles were first viewed in B-mode with the probe placed in the transverse plane to help identify the optimal location. For the MF, the probe was positioned lateral to the L4 spinous process over the L4-L5 zygapophyseal joint. Additionally, it was rotated 10–20° counter-clockwise (measurement of right side) and tilted slightly laterally. For the ES, the probe was located lateral to the L4 spinous process just above the iliac crest at the most prominent part of the muscle belly. The probe was oriented parallel to the spine’s longitudinal axis and slightly tilted to enable a clear image. The probe placement was considered correct when both the superficial fascia and muscle fibers were continuous and uninterrupted on the image. The marks drawn on participants are shown in Figure 2.

**Figure 3:**
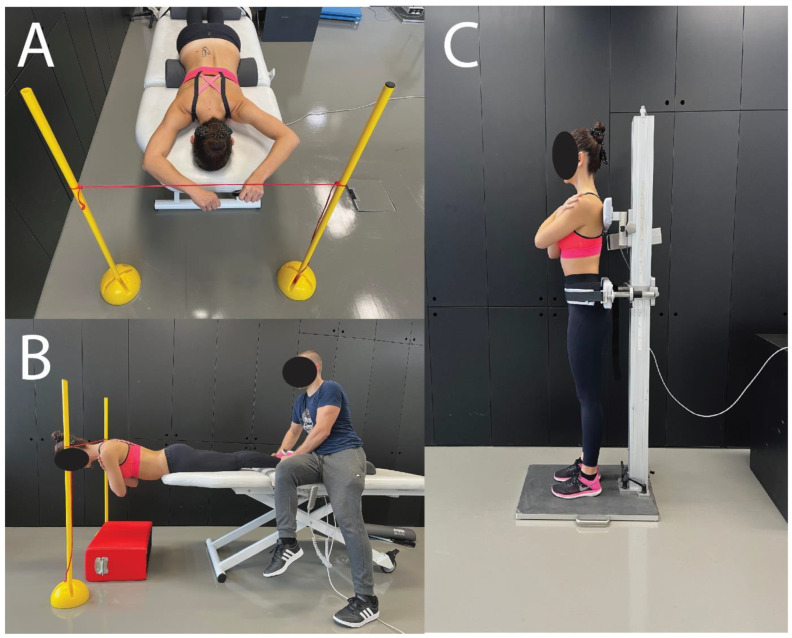
Participant positioning for low-level (**A**) and high-level contractions (**B**) and set-up for isometric trunk extension force measurements (**C**). Ultrasound measurements are not shown in figures A and B to allow clearer representation of the participant positions.

Measurements of shear modulus were completed at rest and during two submaximal contractions of the trunk extensors at different intensities: the prone arm lift (low-level contraction; Figure 3A) and trunk extension (high-level contraction Figure 3B). For the former, participants laid prone with their legs supported on a pillow. Furthermore, a small pillow was placed under the participants pelvis to rotate it posteriorly and flatten the lumbar curve. For the prone arm lift (low-level contraction), the position was the same as during rest. The shoulders were abducted to 120°, and the elbows were flexed at 90°. The lower legs were fixated using a strap belt to prevent lower limb movement during the arm lift. The participants were instructed to lift the contralateral arm while holding a 0.5 kg plate approximately 5 cm above the table and holding this position during the measurement. This procedure has been used before and produced highly reliable shear modulus values [14]. For trunk extension (high-level contraction), we used the Biering-Sorensen test position, as used in previous studies [36]. The participants were positioned with their trunk over the edge of the table, while the pelvis and legs were manually fixated on the table by the researcher. For reference, the superior anterior iliac spines were positioned above the edge of the table. The participants were instructed to lift the upper body by extending the hip to a neutral position (0° hip extension) with their arms crossed on their chest and holding this position. During both submaximal contractions, a thin rope was used for external reference to enable the standardization of measurement at different time points.

All measurements were conducted on the dominant side only (i.e., self-reported side corresponding to the arm the participant would throw a ball). Prior to the warm-up, we measured only muscle stiffness at rest. Moreover, muscle stiffness during submaximal contractions was assessed only for the superficial MF and ES. For the former, the depth of the region of interest was modified in some cases due to the appearance of connective tissue on the image. Nevertheless, the region of interest depth was maintained constant between the two sets of measurements. Deep multifidus stiffness was not evaluated during contraction, as we found highly variable and inconsistent values during the pilot measurements. The location and size of the region of interest was adjusted individually for each participant so as much muscle tissue as possible was covered without including connective tissue. While performing the measurement, the probe was held in contact with the skin with light pressure without compressing it. A water-soluble gel was added to increase the contact between the probe and the skin. For each muscle, three measurement trials were performed with each trial being comprised of eight consecutive scans. The average value of the three measurement trials (24 scans in total) was calculated and included in the final analysis. Within trials, the probe was kept at the same location and was not moved during consecutive scans. Between trials, the probe was removed and then placed at the same position for the next trial.

### 2.4. Maximal Voluntary Force

MVF was measured prior to and following the isometric trunk extension protocol. Maximal voluntary force (MVF) was measured during maximal voluntary contraction of the trunk extensors on an isometric trunk dynamometer (S2P, Ljubljana, Slovenia) with an integrated force sensor (model PW10AC3-200kg, HBM, Darmstadt, Germany) (Figure 3C). The participants stood on the dynamometer with their arms crossed on their chest and the back facing the foamed support pads. The lower support pad was positioned inferior to the base of the sacrum, while the upper support pad was just inferior to the spine of the scapula. The depth of the lower pad was adjusted to achieve a neutral position of the lumbar spine. The pelvis was firmly fixated using a strapped belt. The participants first performed two familiarization trials with ~50% and ~75% of their maximal perceived effort. They were instructed to gradually develop force by pushing with their upper body on the upper support pad and maintain maximal contraction. The trial was terminated when clear plateau in force was reached (observed by the examiner), which was typically within 3 to 5 s. For the maximal effort trials, the researchers verbally encouraged the participants throughout the whole measurement to ensure maximal exertion. Two maximal effort trials were performed and the mean value [N] of two measurements was included in the final analysis. Additionally, MVF was evaluated between sets of the isometric trunk extension protocol to determine a new reference value for the upcoming sets during the fatiguing protocol (described below).

### 2.5. Isometric Trunk Extension Protocol

Fatigue of the trunk extensors was induced with an intermittent isometric trunk extension protocol, which was adopted from Morel et al. [37] and slightly modified for the purpose of our study. The isometric protocol was completed on the same dynamometer as MVF assessments and with the same positioning as described above. Altogether, participants performed two 6-min sets and one 3-min set of intermittent submaximal trunk extensions. Each set consisted of alternating 6-s exertions and 4-s rest intervals. During the exertion, the participants were required to maintain a constant force at 50% MVF. The relative amount of force was modified from the original 60% [37] to 50% following pilot measurements since we observed some individuals struggled to complete the protocol at 60% MVF. The participants were provided with visual real-time feedback of the exerted and targeted force (50% MVF) and another line at 55%. Prior to the beginning of the protocol, the participants completed a familiarization trial to learn to hold the targeted force. They were instructed to hold the force just above the 50%. In case the force fell below 50%, they were warned by the examiner to increase the force. All participants managed to complete the protocol without dropping below the 50% target. The working sets were interspersed with 2 min of rest. At the end of each resting period between sets, the MVF was re-evaluated (“calibration MVF” on Figure 1) to account for fatigue and to set a new reference value for the upcoming set.

### 2.6. Statistical Analysis

The data are presented as means ± standard deviations. The normality of the data distributions for all variables was verified with the Shapiro–Wilk test (all *p* ≥ 0.095). The reliability of MVF and shear modulus before and after fatigue was evaluated with intra-class correlation coefficient (ICC; single measures, absolute agreement) and typical error (TE) expressed as percentage of the mean. We considered ICC values < 0.5 as indicative of poor reliability, values between 0.5 and 0.75 as moderate reliability, values between 0.75 and 0.9 as good reliability, and values greater than 0.90 as excellent reliability [38]. In addition, reliability was considered acceptable when TE was <10% [39]. We used three-way repeated measures analysis of variance with muscle and fatigue as within-subject factors and sex as between-subject factor to explore the effects of fatigue on shear modulus in different muscles in both sexes. Note, all three muscles were included in the analysis for passive shear modulus, while only ES and superficial MF data were available for active shear moduli. In case of significant main effects or interactions, we used Bonferroni-corrected pair-wise *t*-tests to assess the effect of fatigue in specific muscles for the whole sample and separately in each sex. The effect of fatigue on MVF was assessed with two-way analysis of variance (sex × fatigue) and pairwise *t*-tests. For statistically significant effects of analysis of variance and *t*-tests, partial η^2^ and Cohen’s d were also calculated as measures of effect size. The η^2^ values were considered to indicate no effect (<0.01), small effect (0.01–0.039), medium effect (0.06–0.14), and large effect (>0.14), whereas the effect sizes according to Cohen’s d were considered as trivial (<0.2), small (0.2–0.5), medium (0.5–0.8), and large (>0.8) [40]. The threshold for statistical significance was set at α < 0.05, and all analyses were carried out in SPSS statistical software (version 25.0, IBM, Armonk, NY, USA).

## 3. Results

**Figure 4:**
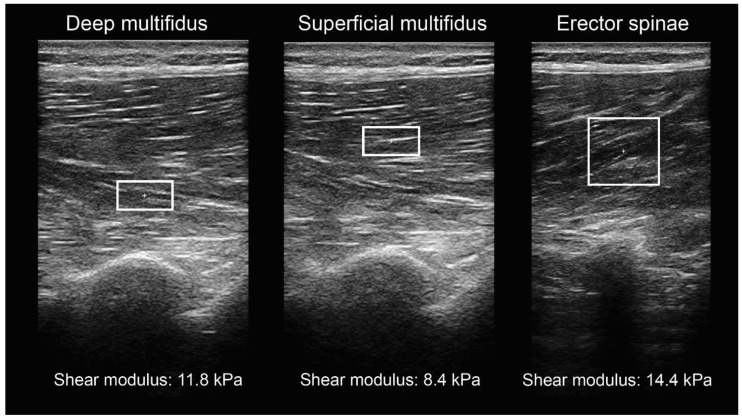
Snapshots from shear modulus measurements at rest. The rectangles represent the region of interest.

The demographic data of the sample were as follows: 22.5 ± 3.0 years of age, 69.3 ± 14.8 kg of body mass, and 172.6 ± 10.2 cm of body height. The average age, body mass, and body height for males were 23.6 ± 3.2 years, 78.5 ± 12.1 kg, and 180.1 ± 5.2 cm. The average age, body mass, and body height for females were 21.4 ± 2.4 years, 60.1 ± 10.7 kg, and 164.8 ± 7.9 cm. Ultrasound snapshots from passive shear modulus measurements are shown in Figure 4.

On a whole-sample level, MVF decreased from 771.2 ± 249.8 N before the fatigue to 707.3 ± 204.1 N after the fatigue (−8.64%; *p* = 0.003; d = 0.71). However, two-way analysis of variance indicated a different response according to sex (*p* = 0.001 and η^2^ = 0.22 for sex × fatigue interaction). Pairwise *t*-test indicated MVF had a statistically significant reduction in both sexes; however, the effect was larger in males (mean difference = −104.8 N (−10.9%); *p* = 0.010; d = 0.55) compared to females (mean difference = −22.9 N (−3.7%); *p* = 0.047; d = 0.17).

The reliability of MVF was excellent before fatigue (ICC = 0.98; TE < 1%) and after fatigue (ICC = 0.97; TE < 1%). Before fatigue, the reliability of passive and active shear modulus values was good to excellent for ES (ICC = 0.82–0.88; TE = 1.53–2.48%), superficial MF (ICC = 0.82–0.92; TE = 1.25–2.73%), and deep MF (ICC = 0.88; TE = 2.80%). In the fatigued state, the reliability was also good to excellent for both passive and active shear modulus for superficial MF (ICC = 0.85–0.91; TE = 1.31–3.91%) and passive shear modulus for deep MF (ICC = 0.89; TE = 2.94%). For the ES muscle after fatigue, the reliability was also good for both active shear modulus outcomes (ICC = 0.85–0.89; TE = 1.72–1.83%). However, only moderate reliability was shown for passive ES shear modulus after fatigue (ICC = 0.63; TE = 4.33%).

The depth of the ROI had a statistically significant difference between measurement conditions for superficial MF (*p* < 0.001; η^2^ = 0.44) (i.e., passive, low-level, and high-level contractions). For the passive measurements, a shallower depth was used (mean 1.87 ± 0.07 cm), which was different from low-level contraction and high-level contraction (both pairwise *p* < 0.001). However, the ROI depth was not different between low-level (2.24 ± 0.11 cm) and high-level level (2.29 ± 0.11 cm) contraction conditions (*p* = 0.545). For the ES muscle, there were no differences between conditions in terms of ROI depth (*p* = 0.112; mean values 2.62 ± 0.40 cm; 2.65 ± 0.39 cm and 2.67 ± 0.43 cm for passive, low-level and high-level contractions, respectively). Sex did not affect depth of ROI for MF (*p* = 0.355) and ES (*p* = 0.851).

### 3.1. The Effects of Warm-Up on Passive Shear Modulus

First, we explored the effect of warm-up on passive shear modulus. The analysis of variance indicated there was no warm-up × muscle interaction (*p* = 0.123) and no effect of warm-up (*p* = 0.783), and a large effect of muscle (*p* < 0.001; η^2^ = 0.44). However, the pairwise test indicated the warm-up had a statistically significant effect on passive shear modulus of ES (−0.61 kPa; *p* = 0.030), while there was no effect in superficial MF (+0.006 kPa; *p* = 0.981) and deep MF (+0.41 kPa; *p* = 0.412).

### 3.2. Changes in Passive Shear Modulus

**Figure 5:**
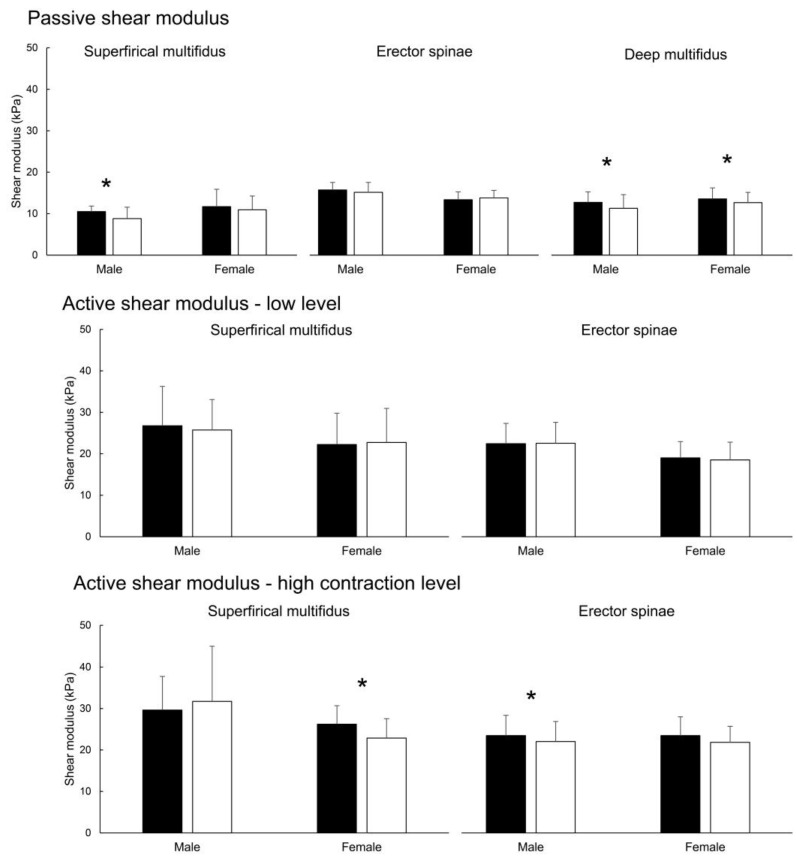
Sex-specific data across muscles and conditions. * Denotes statistically significant pair-wise effect (pre-fatigue vs. post-fatigue).

For the passive shear modulus, there were no muscle × sex × fatigue interaction (*p* = 0.927), no sex × fatigue interaction (*p* = 0.125), and muscle × fatigue interaction (*p* = 0.095). On the other hand, there was a statistically significant main effect of fatigue (*p* = 0.004; η^2^ = 0.33) and muscle (*p* < 0.001; η^2^ = 0.45). Pairwise tests for fatigue effects on a whole sample showed passive shear modulus had a statistically significant decrease after fatigue in superficial MF (mean difference = −1.21 kPa (−11.6%); *p* = 0.022; d = 0.40) and deep MF (mean difference = −1.17 kPa (−11.9%); *p* = 0.006; d = 0.46) but not the ES muscle (*p* = 0.867). Descriptive statistics and pairwise tests are available in Table 1. Additional sex-specific pair-wise tests indicated no changes in ES shear modulus in either sex (*p* = 0.341–0.424) and statistically significant decreases in deep MF (*p* = 0.041–0.048; d = 0.34–0.50) in both sexes. However, passive shear modulus in superficial MF was decreased only in males (*p* = 0.027; d = 0.82), but not in females (*p* = 0.332). Sex-specific data is shown in Figure 5.

### 3.3. Changes in Active Shear Modulus

For the active shear modulus during low-level contraction (only measured for ES and superficial MF), there was no muscle × sex × fatigue interaction (*p* = 0.234), no fatigue × sex interaction (*p* = 0.546), and no fatigue × muscle interaction (*p* = 0.957) nor the main effect of fatigue (*p* = 0.551), while there was a main effect of muscle (*p* = 0.021; η^2^ = 0.23) with higher shear modulus in MF compared to ES. Sex-specific data is shown in Figure 5.

For the active shear modulus during high-level contraction, muscle × sex × fatigue interaction was approaching statistical significance (*p* = 0.051), while there was no muscle × fatigue interaction (*p* = 0.702) nor sex × fatigue interaction (*p* = 0.071). However, there were statistically significant effects of muscle (*p* = 0.001; η^2^ = 0.41) and fatigue (*p* = 0.003; η^2^ = 0.37). Pairwise tests performed on a whole sample indicated a decrease in shear modulus for ES (mean difference = −1.52 kPa (−6.71%); *p* = 0.011; d = 0.34) and superficial MF (mean difference = −1.78 kPa (−6.76%); *p* = 0.011; d = 0.29). The details are available in Table 1. However, sex-specific pair-wise test indicated the decrease in ES shear modulus was statistically significant only in males (*p* = 0.015; d = 0.31) but not in females (*p* = 0.140). Conversely, the shear modulus in superficial MF had a statistically significant decrease in females (*p* = 0.002; d = 0.74) but not in males (*p* = 0.368). Sex-specific data is shown in Figure 5.

### 3.4. Correlations between Changes in Shear Modulus and Maximal Voluntary Force

The relative changes in MVF were not correlated with relative changes in shear modulus in any condition for ES (r = 0.04–0.23; *p* = 0.299–0.860), superficial MF (r = 0.11–0.29; *p* = 0.119–0.612), and deep MF (r = 0.16; *p* = 0.481). When correlations were done specifically for each sex, there was a trend for an association between reduction in MVF and reduction in shear modulus for superficial MF in females (r = 0.51; *p* = 0.061).

## 4. Discussion

The aim of the present study was to investigate the effects of isometric fatigue on the passive and active shear moduli of trunk extensor muscles. Our hypothesis was partially confirmed as fatigue resulted in decreased active shear modulus during high- but not low-level contraction, whereas the effect on passive shear modulus was seen in both the superficial and deep layers of MF but not ES. These results have important implications for further investigations of the mechanistic interaction between physical workloads, muscle stiffness (and other variables affecting trunk stability and neuromuscular control), and the development/persistence of LBP.

Our fatigue protocol caused a ~12% decrease in passive shear modulus for the MF muscle and a ~7% decrease in active shear modulus during high-level contraction for both ES and MF. Our results are consistent with studies performed on lower limb muscles. For example, Siracusa et al. [27] reported a 34% decrease in passive shear modulus in the vastus lateralis following fatiguing contractions resulting in a 38% decrease in MVF. Chalchat et al. [26] recently reported an even greater decrease in vastus lateralis shear modulus (~58%) with a similar reduction in MVF (~37%). Morel et al. [37] observed a smaller decrease in vastus lateralis shear modulus (~17%), which was associated with a smaller decrease in MVF (~22%) compared with other studies. In addition, Mendes et al. [28] observed a progressive decrease in shear modulus of the semimembranosus (but not the biceps femoris) during sustained isometric contraction at 20% of MVF, suggesting load redistribution between the muscles may be a possible factor in the changes in shear modulus. However, in the previously mentioned study by Morel et al. [37], a 38% reduction in shear modulus of the abductor digiti minimi muscle (with ~32% reduction in MVF) was also observed after fatigue. Since this muscle is the only synergist for little finger abduction, their results indicate the reduction in shear modulus observed with fatigue is not exclusively due to load redistribution. In general, our results are consistent with those of previous studies, whereas the discrepancy between the magnitudes of shear modulus reductions could be due to different levels of fatigue, participant characteristics, and muscle groups studied. In further sections, we discuss the possible mechanisms underlying our results.

Studies have shown the shear modulus determined by SWE is consistent with Young’s modulus determined by direct testing (i.e., measuring the relationship between tension and strain) in an isolated muscle under both passive [41] and active conditions [42]. However, several other factors could influence the shear modulus during in vivo human measurements; thus, the decrease observed in our study could be explained in many different ways. As mentioned previously, SWE studies in leg muscles have shown load distribution between synergistic muscles can change with contraction level [43] and fatigue [28]. In addition, there is evidence MF fatigues faster than ES during isometric trunk extension tasks [44]. However, since both MF and ES showed a decrease in shear modulus during high-level contraction, we believe load distribution had a small or negligible effect on our results. Another possible explanation is prolonged isometric contraction triggered a creep response in the tendon [45], resulting in a shorter muscle length and a decrease in shear modulus [41,46]. However, given the relatively short tendons in MF and ES relative to the overall length of the muscle-tendon complex, this also seems an unlikely explanation. Muscle fiber type could also play a significant role as slow-twitch fibers are stiffer than fast-twitch fibers [47,48]. It could be that slow-twitch fibers were fatigued to a greater extent than fast-twitch fibers; this could cause a decrease in active shear modulus as more fast-twitch fibers would be recruited during contraction. However, this would not explain why passive shear modulus was also decreased.

At this point, it should be noted muscle fiber stiffness can change independently of changes in muscle tension [42]. Although muscle stiffness and muscle tension are strongly correlated, they may respond differently to fatigue [49]. This suggests other explanations for decreased stiffness possibly occurring independently of muscle tension changes should be considered. Our protocol included a standardized warm-up prior to baseline measurements to minimize the influence of muscle temperature. In addition, we observed no effect of warm-up on passive shear modulus. However, it cannot be excluded the fatigue protocol led to a further increase in muscle temperature, which could lead to a decrease in active muscle stiffness and active shear modulus at the same muscle force [42], and passive shear modulus [50]. Indeed, an increase in muscle temperature has been shown to induce the unfolding of myosin and collagen [51], which could contribute to a decrease in passive and active stiffness. Interestingly, we observed a ~7% reduction in active shear modulus after fatigue, which is less than the ~12% reduction observed in a study comparing muscle stiffness at 38 °C and 26 °C [42]. However, intramuscular temperature range in vivo [52] is much smaller than the 12 °C difference induced in the aforementioned study [42], implying the temperature change in our study is unlikely to be the only mechanism for the decrease in shear modulus. As a final possibility, fatigue-induced depletion of Ca^2+^ could reduce the number of attached cross-bridges [49] and reduce noncontractile stiffness associated with titin and other passive structures within the sarcomere [53,54]. In summary, the exact underlying mechanisms of the decrease in shear modulus after fatigue and their relative contributions remain unclear and will be an interesting avenue for future research.

MVF was reduced substantially more in males (10.9%) than females (3.7%). Higher susceptibility to fatigue in males has been reported previously in various muscles [55,56], including trunk extensors [57], but the difference was not as pronounced as in the present study. These differences have been attributed to a higher percentage of slow muscle fibers [58] and lower muscle mass in females [56]. In addition, as the contractions in our study were submaximal, blood flow and muscle oxygenation could play an additional role [59]. Indeed, a previous study on handgrip muscles indicated females had higher mean brachial arterial blood flow and vascular conductance in between intermittent maximal isometric contractions [60]. Furthermore, slightly higher minimal detectable changes for trunk extension MVF were indicated in females compared to males [61]. Altogether, these factors could explain why the reduction in MVF was much smaller in females compared to males. Further research should attempt to match male and female participants by muscle strength and muscle mass to examine this further. In addition to different responses to fatigue in terms of MVF, males and females also showed divergent, muscle-specific results in regard to shear modulus. Namely, the pair-wise test indicated the decrease in ES shear modulus was present only in males (d = 0.31), and only for females in superficial MF (d = 0.74). This could point to a different strategy in terms of muscle contributions. Previous studies showed different trunk muscle activation patterns between sexes during gait [62] and static postures [63], but we are not aware of any studies that would examine sex-specific trunk muscle activation during intermittent submaximal isometric contractions.

Our results, together with previous findings [26,27,28,37], suggest fatigue should be considered when studying the shear modulus and its influence on neuromuscular function and injury risk. Our results also show the reduction in shear modulus is not necessarily proportional to the reduction in MVF, which further complicates the issue. For example, load distribution between the quadriceps muscles and the relationship between passive and active shear modulus have been associated with the development of knee osteoarthritis [64] and patellofemoral pain [30], respectively; however, it is not known whether these findings persist under fatigue conditions. Murillo et al. [16] reported a mean difference of ~3.5 kPa for superficial MF between patients with LBP and asymptomatic controls, while Masaki [15] reported even smaller (~0.8 kPa), but statistically significant differences. Considering our findings, researchers should be aware trunk muscle fatigue alone may lead to a decrease in muscle stiffness.

It has been suggested the increase in muscle stiffness in LBP may reflect chronic adaptation to changes in contractile function and muscle architecture [65]. To date, the causal relationship between stiffness changes and LBP has not been established. To this end, studies examining the interplay between physical loading, trunk neuromuscular function, and trunk muscle architectural and mechanical properties (including stiffness) are warranted. However, our results suggest careful study designs are needed to distinguish responses in muscle stiffness (shear modulus) caused by fatigue and other factors (e.g., particular posture, vibration, etc.). It has already been shown changes in trunk neuromuscular control after prolonged trunk flexion are mainly due to tissue creep response rather than fatigue [35], but further carefully conducted experiments are needed to investigate the effects of different physical loads on shear modulus and to distinguish the contribution of fatigue. This is particularly important with respect to the design of interventions. For example, if the detrimental effect of prolonged work in a flexed trunk posture is due to fatigue of the extensor muscles, mechanical support to relieve the muscles might be the most important consideration. If, on the other hand, the posture itself (independent of fatigue) is the cause of the harmful effects, a different approach may be needed. In addition, caution should be exercised when comparing absolute shear modulus values across studies as different ultrasound devices have been shown to produce systematically different outcomes [66].

Some limitations of the present study must be acknowledged. Shear modulus was measured only in an approximately neutral position of the spine. Changes in shear modulus could be specific to muscle length as has been shown after eccentric exercise [67,68]. Furthermore, due to the lack of intramuscular temperature data and shear modulus measurements at multiple spinal segments, we cannot confirm or rule out temperature changes or altered load distribution as underlying mechanisms. In addition, ROI depth had to be altered between conditions to ensure the same portion of each muscle was measured. While it has to be acknowledged ROI depth may influence shear modulus quantification [69], it should not have affected the assessment of fatigue effects as the comparisons were made only within the same condition (passive, low-level, and high-level contractions) with depth of the ROI unchanged. The study sample consisted of healthy adults. It remains unknown whether patients with LBP would respond differently to the fatigue protocol. Finally, we used only MVF as an indicator of fatigue, which is insufficient to distinguish between central and peripheral factors. Previous studies conducted on knee extensor muscles used maximal effort contractions (total time contracting = 300 s) and reported a more notable reduction in MVF (~37–38%) [26,27] alongside reduced evoked torque (~37%). It seems our protocol, which involved contractions performed at 50% MVF, although being longer (total time contracting = 540 s), failed to induce the same level of fatigue reported in the aforementioned study. However, previous studies have observed noticeable fatigue (i.e., decreased mean EMG power frequency) for trunk extensors even during very small levels of sustained contractions (2% of maximal EMG activity) [70]. Therefore, we are confident we have produced meaningful levels of fatigue. In addition, an intermittent submaximal protocol was chosen to better replicate real-life situations. Moreover, we have to acknowledge a learning effect could have occurred for MVF assessment [61]. Nevertheless, in any case, these results have the same relevance for LBP research in any case; repeated submaximal isometric contractions may reduce the shear modulus, even if they are associated with only a low degree of fatigue.

## 5. Conclusions

This study suggests passive and active shear moduli in trunk extensor muscles can be reduced by isometric fatigue. Although the mechanisms underlying these changes remain to be elucidated, our results suggest fatigue should be considered as a confounding factor when studying the effects of physical loading on muscle stiffness. In addition, some sex-specific responses were observed, suggesting sex differences should also be considered in this type of research.

## Figures and Tables

**Table 1:** The comparison of shear modulus scores before and after the fatiguing protocol.

Muscle	Condition	Before Fatigue		After Fatigue		Mean Difference		*t*-Test		
		Mean	SD	Mean	SD	Raw (kPa)	Relative (%)	*t*-Value	*p*	d
Erector spinae	Passive	14.54	2.18	14.47	2.19	−0.07	−0.45	0.17	0.867	0.03
	LC	20.73	4.66	20.53	5.03	−0.20	−0.99	0.39	0.701	0.04
	HC	23.47	4.61	21.94	4.26	−1.53	−6.74	2.80	0.011	0.34
Superficial MF	Passive	11.10	3.09	9.88	3.18	−1.22	−11.62	2.48	0.022	0.39
	LC	24.50	8.69	24.24	7.75	−0.25	−1.04	0.39	0.697	0.03
	HC	27.23	5.89	25.44	6.38	−1.78	−6.77	2.79	0.011	0.29
Deep MF	Passive	13.15	2.56	11.97	2.92	−1.18	−9.36	3.07	0.006	0.46

MF—muflitidus; SD—standard deviation; d—effect size (Cohen’s d); LC—low-level contraction; HC—high-level contraction.

## Data Availability

Not applicable.
